# *Clostridium difficile* flagella induce a pro-inflammatory response in intestinal epithelium of mice in cooperation with toxins

**DOI:** 10.1038/s41598-017-03621-z

**Published:** 2017-06-12

**Authors:** Jameel Batah, Hussein Kobeissy, Phuong Trang Bui Pham, Cécile Denève-Larrazet, Sarah Kuehne, Anne Collignon, Claire Janoir-Jouveshomme, Jean-Christophe Marvaud, Imad Kansau

**Affiliations:** 1 Faculté de Pharmacie, “Unité Bactéries Pathogènes et Santé” (UBaPS), Université Paris-Sud, Université Paris-Saclay, 92296 Châtenay-Malabry Cedex, France; 2 School of Dentistry, College of Medical and Dental Sciences, The University of Birmingham, Birmingham, B5 7EG UK

## Abstract

*Clostridium difficile* is the most important enteropathogen involved in gut nosocomial post-antibiotic infections. The emergence of hypervirulent strains has contributed to increased mortality and morbidity of CDI. The *C. difficile* toxins contribute directly to CDI-associated lesions of the gut, but other bacterial factors are needed for the bacteria to adhere and colonize the intestinal epithelium. The *C. difficile* flagella, which confer motility and chemotaxis for successful intestinal colonization, could play an additional role in bacterial pathogenesis by contributing to the inflammatory response of the host and mucosal injury. Indeed, by activating the TLR5, flagella can elicit activation of the MAPK and NF-κB cascades of cell signaling, leading to the secretion of pro-inflammatory cytokines. In the current study, we demonstrate, by using an animal model of CDI, a synergic effect of flagella and toxins in eliciting an inflammatory mucosal response. In this model, the absence of flagella dramatically decreases the degree of mucosal inflammation in mice and the sole presence of toxins without flagella was not enough to elicit epithelial lesions. These results highlight the important role of *C. difficile* flagella in eliciting mucosal lesions as long as the toxins exert their action on the epithelium.

## Introduction

The Gram-positive anaerobic bacterium *Clostridium difficile* is responsible for intestinal nosocomial post-antibiotic infections in developed countries. The clinical features of *C*. *difficile* infection (CDI) include diarrhea, moderately serious disease, and severe pseudomembranous colitis. The major risk factors associated with CDI are antibiotic exposure, hospitalization, and advanced aged^1^. A dramatic increase of severe disease and mortality of CDI have been observed in North America, Europe and Australia^2, 3^, mainly resulting from the emergence of highly virulent and epidemic *C. difficile* strains^3, 4^.

The *C. difficile* toxins TcdA and TcdB are largely involved in lesions of the gut observed during CDI^5, 6^, but other factors such as adhesins^7–10^, hydrolytic enzymes^11, 12^, the S-layer proteins^13^, and cell wall proteins^9^, are needed for the bacteria to adhere and colonize the gut. The *C. difficile* flagella confer motility and chemotaxis for successful intestinal colonization following disruption of the bacterial microbiota^14, 15^. However, flagella could play an additional role in bacterial pathogenesis by contributing to the inflammatory response of the host and mucosal injury. Indeed, flagellin, the principal component of bacterial flagella, is recognized by the Toll-like receptor 5 (TLR5)^16^, one of the Pattern Recognition Receptor (PRR) involved in innate immune response, which is mostly localized at the basolateral pole of intestinal cells. The TLR5-flagellin interaction triggers activation of the MAPK and NF-κB cascades of cell signaling, leading to the secretion of pro-inflammatory cytokines^17, 18^.

To date, few studies have addressed this role for *C. difficile* flagella. Yoshino *et al*. showed that *C. difficile* flagellin induces activation of NF-κB and the p38 MAPK, thus promoting the synthesis of IL-8 and CCL20 in intestinal epithelial cells^19^. These authors showed that a pretreatment with toxin TcdB enhances the flagellin-induced cytokine production by cells. Recently, we reported that the interaction of *C. difficile* flagellin and TLR5 predominantly activates the NF-κB pathway, thus leading to up-regulation of pro-inflammatory gene expression and subsequent synthesis of pro-inflammatory mediators^20^.

The aim of the current study was to evaluate the role of *C. difficile* flagella in cooperation with toxins in eliciting an inflammatory host response during *in vivo* infection. By using a conventional mouse model of CDI and different *C. difficile* mutants lacking flagella or toxins, we observed that the absence of flagella dramatically decreases the degree of mucosal inflammation in mice and the sole presence of toxins without flagella was not enough to elicit epithelial lesions as observed in mice infected with wild-type bacteria. These results highlight the important role of *C. difficile* flagella in eliciting mucosal lesions as long as the toxins exert their action on the epithelium.

## Results

### Toxigenic and flagellated *C. difficile* R20291 strain, in contrast to non-flagellated or non-toxigenic strains, induce a caecal inflammatory response in the CDI mouse model

**Figure 1:**
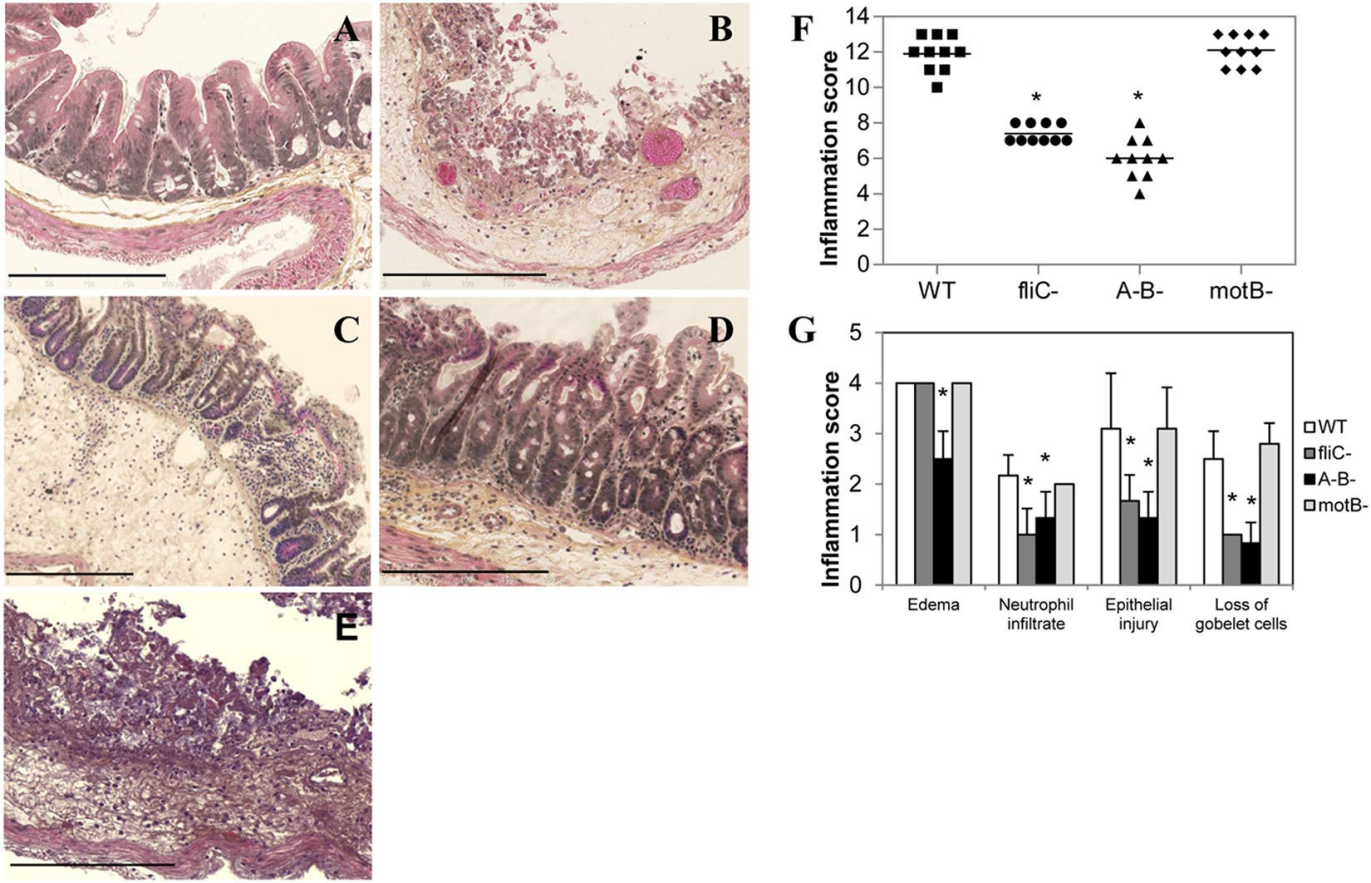
*C. difficile* R20291 flagella are involved in caecal inflammation of mice. C57BL/6 mice (n = 10) were infected or not and caeca were collected at the clinical end point day 2 for histology. (**A**) Representative histology of caecum of healthy uninfected mice (control) with epithelium integrity; (**B**) R20291 WT-infected mice with ulcerative colitis, necrosis, desquamation, exudates and necrotic cells in the intestinal lumen, edema, inflammatory submucosal cell infiltration, loss of architecture of epithelium and presence of pseudomembranes in caecum; (**C**) R20291 *fliC* and (**D**) A^−^B^−^ mutant-infected mice with edema, focal desquamation of necrotic cells in the intestinal lumen and submucosal cell infiltrate; (**E**) R20291 *motB* mutant-infected mice with similar degree of mucosal lesion than R20291 WT-infected mice (200X magnification). Bar = 200 µm. (**F**) Inflammation histological scores for individual mice in each group. The horizontal lines represent the mean scores for each group of animals. (**G**) Individual evaluation criteria of intestinal inflammation. The bars represent the mean scores for each group of animals and standard deviations. *P < 0.01 compared to WT-infected mice.

To study the role of *C. difficile* flagella in the intestinal inflammatory response *in vivo*, we used a CDI model in conventional mice^21^. As expected, all mice infected with the hypervirulent WT R20291 strain (n = 10) developed CDI with diarrhea on the first day post-infection and showed ruffled fur and reduced activity at day 2 after challenge, with a 50% (6/12) mortality rate from day 2 post-challenge. A strong colitis with significant caecal dilatation, luminal liquid accumulation and wall hyperemia was observed in all WT-infected mice. To measure the degree of inflammation of the caecal mucosa of mice, a histological score based on 4 criteria (submucosa edema, inflammatory cell infiltrate, epithelial injury and loss of goblet cells) was developed (Supplementary Table S1). Indeed, a high inflammation score of 12 out of 15 was observed in caecal sections of WT R20291-infected mice compared to uninfected mice (Fig. 1A,B).

In contrast, mice infected with the *fliC* (unflagellated but toxigenic) mutant (n = 10) presented only soft stools at day 1 or 2 post-infection, and survived the challenge. Similarly, the animals infected with flagellated but non-toxigenic A^−^B^−^ mutant (n = 10) did not develop CDI and all animals survived at day 2 post-infection. A mild colitis without caecal dilatation or wall hyperemia was observed in the *fliC* or A^−^B^−^ mutant-infected mice, compared to the group of non-infected mice (n = 10), suggesting that both TcdA/TcdB toxins and flagella are necessary to induce CDI in this mice model. Moreover, a slight inflammation with moderate edema, focal erosion, mild luminal cell desquamation and minimal loss of goblet cells was observed in the *fliC* or A^−^B^−^ mutant-infected mice (Fig. 1C,D). A statistically significant difference (P < 0.01) was observed between the score of the WT-infected mice group (score 12) compared to that of the other two groups (*fliC* and A^−^B^−^ mutant-infected mice, scores 8 and 6 respectively) and control (score 0, Fig. 1F). The analysis of each inflammation criteria separately also showed (except for edema) a statistically significant difference (P < 0.01) between the WT-infected mice and the other two groups (Fig. 1G).

### Toxigenic and flagellum-paralyzed *C. difficile* R20291 mutant induces a caecal inflammatory response in mice

In order to test the role of flagellum in the epithelial inflammatory response we also performed *in vivo* experiments using a *motB* mutant in R20291 strain which is toxigenic and flagellated but not mobile. The *motB* gene is involved in synthesis of the flagellar motor. Interestingly, all mice infected with the paralyzed flagella *motB* mutant (n = 10) developed CDI with a high (50%) mortality rate, a strong colitis with significant caecal dilatation, luminal liquid accumulation and wall hyperemia as observed in the WT R20291-infected mice. Indeed, as for WT R20291-infected mice, mice infected with the flagella paralyzed *motB* mutant showed the highest inflammation score (12 out of 15) in caecal sections compared to uninfected mice (Fig. 1E), with a statistically significant difference (P < 0.01) between the score of the *motB* mutant-infected mice group (score 12) and those of the other two groups (*fliC* and A^−^B^−^ mutant-infected mice, scores 8 and 6 respectively) and control (score 0, Fig. 1F). The individual inflammation criteria were similar to those of the WT-infected mice and a statistically significant difference (P < 0.01) between the *motB*-infected mice and the other two groups was observed (Fig. 1G).

To note, in all performed experiments, the differences observed between the different groups of animals were not due to differences in the rate of fecal colonization since the fecal shedding of vegetative forms and spores reached by these strains were similar for the 4 groups of mice at days 1 and 2 post-infection (Supplementary Fig. S1A,B). Moreover, as expected, the feces from WT strain, *fliC* and *motB* mutant-infected mice, but not those from A^−^B^−^ mutant-infected mice, showed a strong and similar level of cytotoxic activity (Supplementary Fig. S2A), thus indicating that *in vivo* toxin production of the WT R20291 strain and its respective *fliC* and *motB* mutants are quite comparable.

Taken together, these observations suggest that, in presence of toxins TcdA and TcdB, the flagellum of the *C. difficile* R20291 strain plays an important role in amplifying the intestinal inflammatory response during infection, and that these toxins are necessary for the pro-inflammatory activity of flagella in this CDI animal model.

### Flagella and toxins in non-epidemic *C. difficile* 630Δ*erm* strain are also necessary to induce a caecal inflammatory response in the CDI mouse model

**Figure 2:**
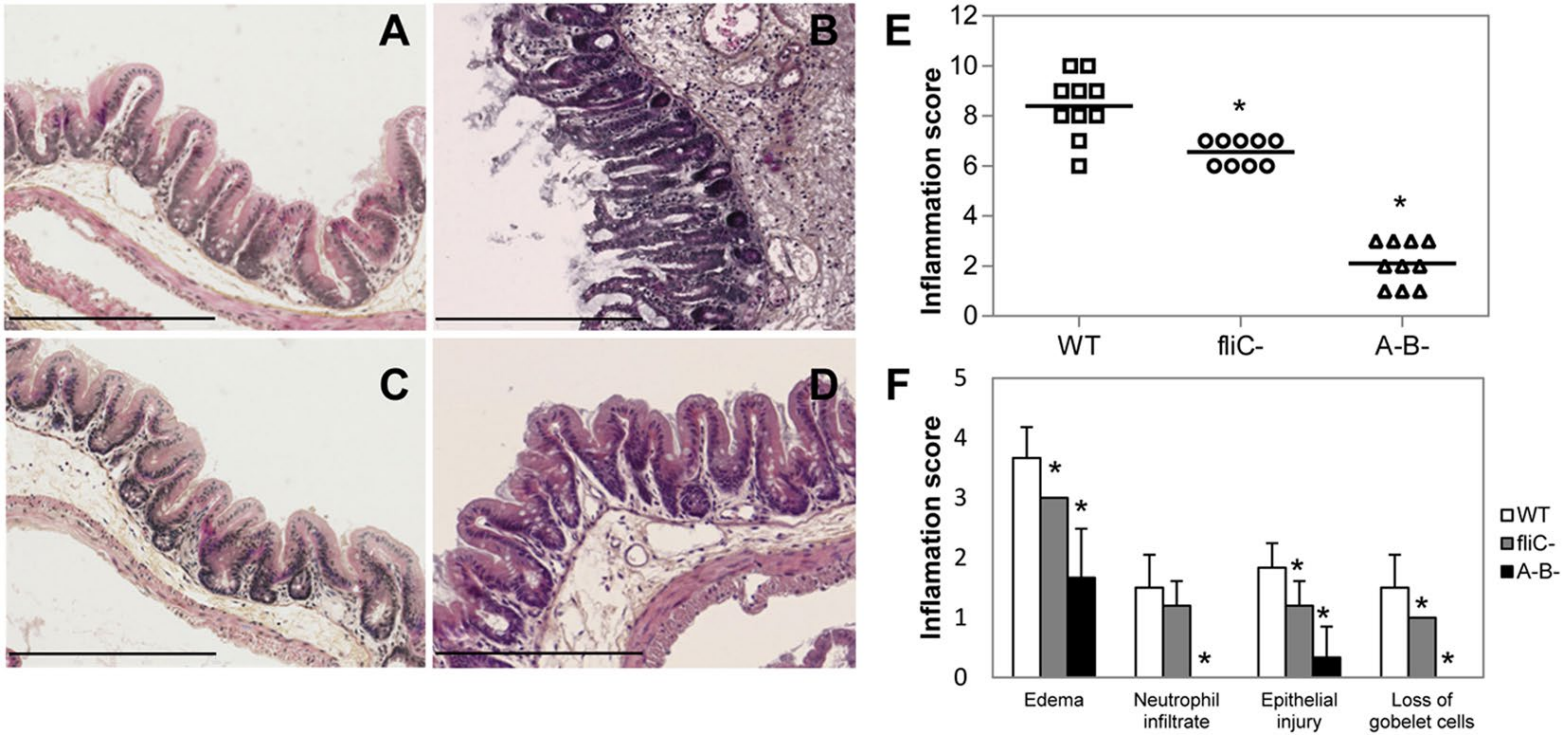
*C. difficile* 630Δ*erm* flagella are involved in caecal inflammation of mice. C57BL/6 mice (n = 10) were infected by oral gavage or not and caeca were collected at the clinical end point day 2 for histology. (**A**) Representative histology of caecum of healthy uninfected mice (control) with epithelium integrity; (**B**) 630Δ*erm* WT-infected mice with moderate desquamation, exudates and necrotic cells in the intestinal lumen, edema, and inflammatory submucosa cell infiltration; (**C**) *fliC* and (**D**) A^−^B^−^ mutant-infected mice with normal mucosa (200X magnification). Bar = 200 µm. (**E**) Inflammation histological scores for individual mice in each group. The horizontal lines represent the mean scores for each group of animals. (**F**) Individual evaluation criteria of intestinal inflammation. The bars represent the mean scores for each group of animals and standard deviations. *P < 0.01 compared to WT-infected mice.

To evaluate a potential strain-dependent effect for FliC-induced caeca inflammation, we performed the same animal experiments using the non-epidemic *C. difficile* 630Δ*erm* strain and its respective *fliC* and A^−^B^−^ mutants. As for R20291 derivatives, comparable fecal shedding was observed for all 630Δ*erm* derivative strains (Supplementary Fig. S1C,D) and a cytotoxic activity was detected in feces of the WT 630Δ*erm*- and the *fliC* mutant-infected mice, while no cytotoxicity was detected in feces of the A^−^B^−^ mutant-infected animals (Supplementary Fig. S2B). In contrast, *in vivo* cytotoxicity was dramatically lower in *C. difficile* WT 630Δ*erm*-infected mice (600 U), compared to that observed in the R20291-infected animals (40 000 U), which is in accordance with the different clinical outcomes observed between these animal sets. Indeed, the flagellated WT 630Δ*erm* induced CDI with soft stools, but a moderated degree of caecal mucosa inflammation compared to uninfected mice (Fig. 2A,B), and the inflammation level was lower (score 8.5, Fig. 2E,F) than that observed in the WT strain R20291-infected mice (score 12). Even if an important submucosa edema was observed, the degree of neutrophil infiltrate, epithelial injury and loss of goblet cells was considerably reduced in caecal sections of 630Δ*erm* WT-infected mice compared to sections of R20291-infected mice. Moreover, as for the *fliC* mutant in R20291, the 630Δ*erm fliC* and A^−^B^−^ mutants did not induce disease and elicited only a weak degree of caecal mucosa inflammation (Fig. 2C,D) as measured by histological scores (Fig. 2E,F). These results suggest that flagella of two different epidemic and non-epidemic *C. difficile* strains contribute with toxins to caecal inflammation during infection in mice, despite major differences in the intensity of the inflammation induced by the two strains.

### The absence of TLR5 strongly reduces the *C. difficile* infection-induced caecal inflammation of mice

**Figure 3:**
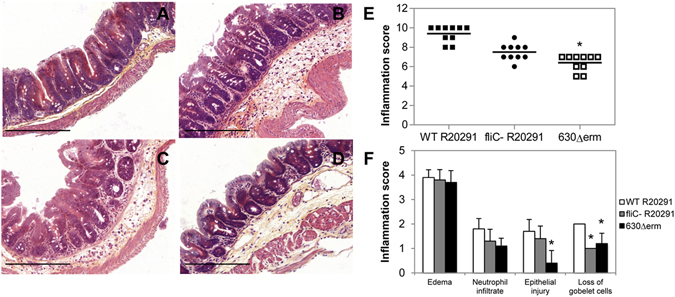
*C. difficile* flagella and caecal inflammation in C57BL/6 *tlr5* ^−*/*−^ KO mice. C57BL/6 *tlr5* ^−/−^ KO mice (n = 10) were infected by oral gavage or not and caeca were collected at the clinical end point day 2 for histology. (**A**) Representative histology of caecum of healthy uninfected mice (control) with epithelium integrity; (**B**) R20291 WT-infected mice with mild to moderate edema; (**C**) R20291 *fliC* mutant and (**D**) 630Δ*erm* WT-infected mice with mild to moderate edema (200X magnification). Bar = 200 µm. (**E**) Inflammation histological scores for individual mice in each group. The horizontal lines represent the mean scores for each group of animals. (**F**) Individual evaluation criteria of intestinal inflammation. The bars represent the mean scores for each group of animals and standard deviations. *P < 0.01 compared to R20291 WT-infected mice.

In order to analyze the role of the mucosal TLR5 in the *C. difficile* flagella-induced inflammatory response, we tested the CDI model in C57BL/6 *tlr5* ^−/−^ KO mice, which do not express TLR5 in the intestinal mucosa. Mice were infected by oral gavage with the *C. difficile* R20291 WT strain (n = 10) or its *fliC* (unflagellated but toxigenic) mutant (n = 10), as well as with the 630Δ*erm* WT strain (n = 10) and the clinical outcome and caecal lesions were analyzed. No infected *tlr5* ^−/−^ KO mice developed CDI and all animals survived at day 2 post-infection regardless of the strain. Neither colitis was observed in infected mice, compared to the group of non-infected mice (n = 10), and only mucosal edema was detected in histological sections from caecum of animals (Fig. 3B–D), probably induced by *C. difficile* TcdA/TcdB toxins, produced by all tested strains and detected in feces of all infected animals (Supplementary Fig. S2C). In accordance with the low level of cytotoxic activity detected in WT 630Δ*erm*-infected mice, the lowest inflammation score was observed in animals infected by this non-epidemic strain. Altogether, these results strongly suggest that *C. difficile* flagella-TLR5 interaction leads to a pro-inflammatory response of the intestinal mucosa.

### The *C. difficile* flagella, in presence of toxins, induce the NF-κB activation in the caeca of mice

**Figure 4:**
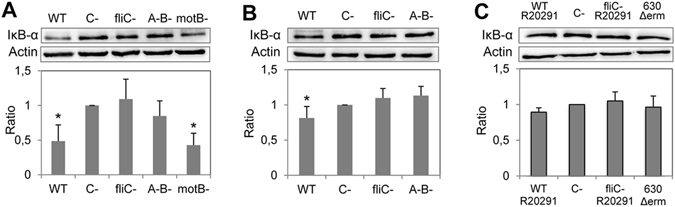
*C. difficile* flagella-induced degradation of IκB-α. Caecal lysates were prepared as indicated in Material and Methods section and proteins were resolved by SDS-PAGE. (**A**) R20291 derivative-infected C57BL/6 mice; (**B**) 630Δ*erm* derivative-infected C57BL/6 mice, and (**C**) R20291 derivative- or 630Δ*erm* WT-infected C57BL/6 *tlr5* ^−/−^ KO mice. Western blot were then performed using anti-IκB-α, actin antibodies. Western blot cropped pictures (full-length blots are in the Supplementary Information file) show the results of a representative experiment. The density of the bands was measured using Fusion software. Ratios IκB-α/actin were calculated and for the negative control (C-, uninfected mice), this ratio was normalized to 1. The ratio of the other samples was reported to the negative control. Results represent the mean (n = 10) ± standard deviations for each group of animals. *Statistically significant differences (P < 0.05) compared to the negative control.

We previously showed that the NF-κB and MAPKs signaling were activated by *C. difficile* flagellin via TLR5 in epithelial cells, with a predominant activation of the former^20^. We thus analyzed the NF-κB activation (IκB-α degradation) in the intestine of mice. Significant IκB-α degradation (2 fold) was observed in the R20291 WT-infected mice compared to the negative control (Fig. 4A). Nevertheless, non-significant IκB-α degradation was observed in the *fliC* and A^−^B^−^ mutant-infected animals, compared to R20291 WT-infected mice (Fig. 4A). Interestingly, as for WT-infected mice, a strong NF-κB activation was observed in caeca of the *motB* mutant-infected mice compared to the negative control (Fig. 4A). We also analyzed the ERK1/2 and JNK MAPKs activation in the intestine of mice. Significant increase of ERK1/2 (1.5 fold) and JNK (2.5 fold) phosphorylation was observed in the R20291 WT- and *motB* mutant-infected mice compared to the negative control (Supplementary Fig. S3A,B). A similar level of ERK1/2 and JNK activation was also observed in the caecum of the *fliC* mutant, but not in the non-toxigenic A^−^B^−^ mutant-infected animals (Supplementary Fig. S3A,B), suggesting a probable action of toxins which are known to also activate MAPKs in intestinal cells^22^. In accordance to the lesser degree of caecal inflammation observed in the 630Δ*erm* WT-infected mice, a weak but significant activation of NF-κB, ERK1/2 and JNK, in caecal tissue of these animals (Fig. 4B and Supplementary Fig. S3C,D). However, no activation of these effectors was observed in caecal tissue from the *fliC* and A^−^B^−^ mutant-infected mice. Moreover, according to the role of TLR5 pro-inflammatory cell-signaling, no NF-κB or MAPK activation was observed in caeca of *tlr5* ^−/−^ KO-infected mice (Fig. 4C and Supplementary Fig. S3E,F). These results are consistent with our previous *in vitro* experiments and suggest that both TcdA/TcdB and flagella are required for NF-κB activation in caeca of *C. difficile* infected mice.

### Toxigenic and flagellated *C. difficile* strains elicit up-regulation of pro-inflammatory cytokine genes in the caeca of mice

Finally, we investigated the changes in the transcription of genes encoding pro-inflammatory cytokines in the intestinal mucosa of *C. difficile*-infected mice. We selected a set of genes whose over-expression was observed previously (Table 1)^20^. As expected, at day 2 post-infection the KC gene encoding keratinocyte derived chemokine (equivalent of the hIL-8 in the mouse) was highly over expressed (114-fold) in the set of R20291 WT-infected mice compared to the control group of non-infected mice (Table 1). A strong up-regulation was also observed for the genes encoding the pro-inflammatory cytokines IL-6 and IL-1β (47- and 49-fold respectively), in accordance to the clinical and histopathological features observed in this group of animals. An up-regulation, but to a lesser extent, was shown for the genes encoding IL-22, TNFα and CXCL-10 (Table 1). Interestingly, in the *fliC* mutant-infected mice, expression of KC (hIL-8) was increased by only 17-fold and the increase of other cytokines (IL-6, IL-1β, IL-22, CXCL-10, and TNFα,) was from 2 to 9-fold, compared to the control group, suggesting a role for the flagellum of the strain R20291 in the development of this pro-inflammatory profile in the gut of infected animals. In the non-toxigenic A^−^B^−^ mutant-infected mice, a lower KC gene up-regulation was also observed (5-fold relative to the control group) as for the other genes, with the exception of the IL -1β encoding gene that showed an over-expression slightly greater than that observed in the *fliC* mutant-infected mice (Table 1). As expected, and according to clinical and histological observations, a strong up-regulation (except for TNFα gene) was also observed for all these pro-inflammatory cytokines encoding genes in animals infected with the paralyzed flagella *motB* mutant (Table 1), strongly suggesting that the coexistence of flagella and toxins largely contributes to the mucosal injury.

**Table 1:** Fold expression of pro-inflammatory cytokine encoding genes of *C. difficil*e-infected mice.

**Set of infected mice**	Genes*					
	**KC**	**IL-6**	**IL-1β**	**IL-22**	**CXCL-10**	**TNFα**
Conventional mice						
R20291 WT strain	113, 5	46, 8	49, 3	5, 7	9, 7	8, 2
R20291 *fliC* ^−^ mutant	17, 5	9, 3	7, 9	3, 7	2, 2	2, 9
R20291 A^−^B^−^ mutant	5, 1	4, 9	10, 0	2, 8	1, 8	1, 6
R20291 *motB* ^−^ mutant	92, 5	39, 0	32, 4	41, 5	18, 0	5, 4
630∆*erm* WT strain	14, 9	8, 3	12, 9	4, 4	3, 5	4, 6
630∆*erm fliC* ^−^ mutant	8, 6	7, 4	7, 9	1, 7	3, 9	3, 9
630∆*erm* A^−^B^−^ mutant	1, 5	1, 7	1, 7	1, 5	1, 7	2, 8
*tlr5* ^−/−^ KO mice						
R20291 WT strain	23, 0	3, 2	11, 1	15, 4	2, 6	6, 0
R20291 *fliC* ^−^ mutant	14, 9	1, 5	1, 0	6, 8	1, 4	1, 0
630∆*erm* WT strain	8, 4	2, 1	8, 7	8, 5	2, 2	3, 0

*The mean Ct from each group is reported to those of the negative control and with the expression of the GAPDH gene for each set.

We next tested the role of flagella from the non-epidemic *C. difficile* 630Δ*erm* strain and its derivative mutants in cytokine gene up-regulation using the CDI mouse model. In accordance with clinical and histopathological observations, the flagellated WT 630Δ*erm* induced a weak up-regulation of KC, IL-6, IL-1β, IL-22, CXCL-10 and TNFα genes compared to the WT R20291 (Table 1). This gene up-regulation was further decreased in the 630Δ*erm fliC* mutant-infected mice and completely null in the 630Δ*erm* A^−^B^−^ mutant-infected animals, except for TNFα. These results indicate that despite differences in virulence between R20291 and 630Δerm *C. difficile* strains, both flagella and toxins contribute to the cytokine gene over-expression from intestinal mucosa in the mouse model, strongly suggesting that toxins are necessary for the contributing pro-inflammatory role of *C. difficile* flagella.

We also analyzed the pro-inflammatory cytokines gene transcription in the intestinal mucosa of the *C. difficile-*infected *tlr5* ^−/−^ KO mice. The infection with the R20291 WT strain elicited a 23-fold up-regulation of KC expression compared to non-infected KO mice, which was very similar to the level of up-regulation observed in R20291 *fliC* mutant-infected conventional mice (Table 1). These results strengthen the role of flagella-TLR5 interaction in the intestinal pro-inflammatory response of the CDI animal model. A lesser up-regulation of this gene was also observed in the R20291 *fliC* mutant- and 630∆*erm* WT-infected *tlr5* ^−/−^ KO mice (Table 1), thus suggesting that toxins, and probably other bacterial products, elicit up-regulation of pro-inflammatory cytokines genes, independently of flagella in *tlr5* ^−/−^ KO mouse model.

## Discussion

Bacterial flagella can be involved in the mucosal inflammatory injury^23^. Indeed, although the surface components of bacteria in the intestinal microbiota play a role in modulating the adaptive immune response, some of these components, including flagella, from pathogenic bacteria can reach the mucosa and play a role in amplifying the inflammatory response. Flagella from some pathogens such as *Salmonella* and *Pseudomonas aeruginosa*, by specifically recognizing the innate immune pattern-recognition receptor TLR5, which is expressed at the basolateral pole of the intestinal epithelium, are responsible for inflammatory lesions in the mucosal epithelium during infection^18, 24^. The bacterial flagellins-TLR5 interaction elicits the MAPKs and NF-κB TLR5-related signaling pathways, thus inducing a pro-inflammatory response from host^16^.

The role of *C. difficile* flagella in the gut inflammatory response remains to be demonstrated. The predicted TLR5-agonist of *C. difficile* flagellin, which shares the conserved amino acids recognized by TLR5 with other mucosal pathogens, but not with flagellins known to evade TLR5^25, 26^, was previously confirmed by studies from Yoshino *et al*. and ourselves^19, 20^. We observed a predominant role for NF-κB, accounting for the importance of this major transcription factor in the *C. difficile* flagellin-associated pathogenesis^27^.

*C*. *difficile* toxins have been considered as the only pathogenic factors involved in intestinal lesions observed during CDI, and several studies have shown their role in stimulating pro-inflammatory signaling pathways^28^. The first evidence of the contribution of *C. difficile* TcdB to the flagellin-induced inflammatory activity was reported by Yoshino *et al*.^19^. They hypothesize that toxin B by affecting tight junctions facilitate the access of flagellin to its receptor in polarized cell monolayers. In a recent report, Kasendra *et al*.^29^ suggest that *C. difficile* toxins facilitate bacterial colonization by disrupting cell polarity allowing pathogen-associated molecular patterns (PAMPs) to access the exposed basolateral epithelial surface and trigger the production of inflammatory cytokines. Nevertheless, the synergy between toxins and other bacterial compounds involved in inflammation has never been demonstrated *in vivo*. For the first time, we show the putative role of flagella in *C. difficile* pathogenesis in a CDI mouse model^21^. In this model, we reproduced pathogenesis observed in human CDI as well as different clinical outcomes depending on two distinct clinical strains and demonstrated that the absence of flagella in toxin producing bacteria dramatically decreases the degree of mucosal inflammation in mice.

We previously reported no difference in the *in vitro* toxin gene expression between the *fliC*-R20291 mutant and its respective wild-type strain, but increased toxicity in the *fliC*-630∆erm flagella mutant compared to its respective WT strain^30^. In agreement with these results, our previous *in vivo* transcriptomic analysis performed in the gnotobiotic mouse model, revealed no or weak differences in the toxin gene expression between this *fliC*-R20291 mutant and its parental wild-type strain^31^. Therefore, toxin gene regulation in absence of FliC is quite different between strains 630 and R20291 and the high mortality previously observed in the monoxenic mice infected by the *fliC*-R20291 mutant might be explained not by an increase in toxin gene expression but by the regulation of other genes whose expression is dependent on flagella^31^. In the present work, we used a different mouse model, the conventional mouse model^21^, much closer to the human CDI, in which both R20291 and 630∆erm strains and their respective *fliC* mutants induced similar cytotoxicity titer levels in feces.

In this study, the presence of toxins without the flagella was not enough to elicit the high degree of epithelial lesions observed in WT R20291-infected mice. The absence of flagella or motility could prevent the *fliC* R20291 mutant to reach the mucosa and thus to produce inflammatory lesions induced by toxins alone. However, in our study, we found levels of fecal colonization and cytotoxic activity in feces in the unflagellated (*fliC*) and non-motile (*motB*) mutant-infected mice, which were similar to those observed in the WT R20291-infected mice, thus reflecting successful intestinal colonization by these flagellar mutants in this CDI model. Moreover, the flagella-paralyzed *motB* mutant was not only able to colonize the mucosa, but also to produce toxins and elicit mucosal lesions as the WT strain. Therefore, in the context of the natural CDI, even if other bacterial factors, bacterial fitness or metabolic adaptation capability could play a role in the pathogenesis, flagella highly contribute to the development of a detrimental inflammatory host response and the outcome of CDI as long as toxins exert their action on the epithelium. Interestingly, this is not the case of cholera, which is the archetypal non-inflammatory diarrheal disease. In *V. cholerae* infection no synergy should occur between flagella and cholera toxin, which induces non-inflammatory watery diarrhea. In the case of CDI, it seems more evident that toxins, by eliciting their actions on the cell cytoskeleton and epithelial tight junctions, could promote the interaction between the FliC monomers and the newly exposed TLR5 and the subsequent pro-inflammatory epithelial host response. According to this role of *C. difficile* flagella, analysis of the CDI elicited by R20291 and 630Δ*erm C. difficile* strains in the *tlr5* ^−/−^ KO mice, which do not express TLR5 in the intestinal mucosa, strengthens the role of the TLR5-related flagellar signaling in the pathogenesis of *C. difficile*. Indeed, mice infected by the epidemic R20291 or the non-epidemic 630Δ*erm* strains developed only mucosal edema with a very low inflammation score, without clinical signs of CDI. This mucosal edema was probably induced by TcdA/TcdB toxins, which were produced by all the tested strains.

We previously compared the role of R20291 and 630Δ*erm C. difficile* strains in adhesion and colonization *in vitro* and *in vivo* and showed major behavioral differences between these strains in a germ-free (gnotoxenic) mouse model^30^. In the current study, we show another important role for *C. difficile* flagella in eliciting an inflammatory host response shared by both strains, but according to the hypervirulent traits of strain R20291, differences persist in the degree of elicited response which may be linked to the level of *in vivo* regulation of toxin synthesis. Nevertheless, in the two strains, toxins alone were not enough to induce the clinical manifestations and the mucosal lesions observed in this CDI mouse model. Considering the high homology in TLR5-recognizing motifs in FliC from the two strains, we can suggest a similar role for flagella from the two strains in eliciting a pro-inflammatory immune response during CDI.

Our work contributes to the knowledge about the virulence factors and pathogenesis of *C. difficile* and highlights other bacterial and host targets in the control of CDI. *C. difficile* flagellin might constitute an interesting vaccine candidate leading to a protective immune response against intestinal pathogens. Interestingly, purified *Salmonella* Typhimurium-derived flagellin protects mice from CDI^32^. In this context, by stimulating TLR5, flagellin could contribute to the bacterial clearance. On the other hand, the outcome of the CDI could be modulated by targeting the pro-inflammatory signaling pathways so as to reduce the severity of the lesions induced by a strong inflammatory response. Thereby, by deciphering the regulation mechanisms of the TLR5 signaling cascade, it would be possible to target potential effectors of the pro-inflammatory response in order to reduce the effects of a deleterious response.

Altogether, our results strongly suggest the role of the *C. difficile* flagella-TLR5 interaction leading to a pro-inflammatory response of the intestinal mucosa in synergy with TcdA and TcdB. Therefore, the *C. difficile* flagellin would exert its action on the pivotal NF-κB signaling pathway through exposed TLR5, thus resulting in the development of a deleterious innate immune response which contributes to the pathogenesis of this intestinal pathogen.

## Materials and Methods

### Bacterial strains and growth conditions

The *C. difficile* strains used in this study were NAP1/027 R20291 wild-type strain (WT), the *fliC* and the *motB* flagellar mutants^30^, and the *tcdA* and *tcdB* double mutant (A^−^B^−^)^33^, and the respective *fliC*-complemented strain (containing pMTL-SB1 *fliC*-complementation plasmid) as described previously. These mutants were created from *C. difficile* R20291 by insertional inactivation using the ClosTron gene knock-out system. For some experiments *C. difficile* 630Δ*erm* strain and its *fliC* ^30^ and A^−^B^−^^6^ isogenic mutants (ClosTron) were also used. *C. difficile* strains were cultured in Brain Heart Infusion (BHI) agar or broth (Oxoid) in an anaerobic chamber (atmosphere of 90% N_2_, 5% CO_2_ and 5% H_2_) at 37 °C. Strains containing the pMTL plasmid were grown in BHI supplemented with 15 µg/ml thiamphenicol. *Escherichia coli* Top10 and BL21 were cultured in Luria-Bertani agar or broth (Oxoid) supplemented with 50 µg/ml kanamycin.

### *C. difficile* spores for mouse challenge

*C. difficile* spores were prepared at 37 °C in an anaerobic chamber as previously described by Burns *et al*.^34^. Briefly, a preculture was grown overnight in BHI supplemented with yeast extract (5 mg/ml, BD) and L-cysteine (0.1%, Merck) (BHIS) and was used to inoculate a starter preculture in BHIS, which was grown to OD_600nm_ 0.2–0.5. Sporulation was then induced in BHIS by inoculation with the starter culture (1 in 100) and incubation for 7 days. Cultures were then washed in sterile water and heated at 70 °C for 25 min to kill vegetative cells and collect spores. Enumeration of spores was performed on BHI agar supplemented with the bile salt taurocholate 0.1% (Sigma-Aldrich).

### Animal model

6–7 week old female C57BL/6 mice were purchased from Charles River (France). 6–7 week old female C57BL/6 *tlr5* ^−/−^ knock-out (KO) mice were kindly provided by Thierry Pedron (Philippe Sansonetti laboratory, Pasteur Institute, Paris) and were housed and bred at the animal facility of the Faculty of Pharmacy, Paris-Sud University. Mice were housed in groups of 5 in sterile cages containing irradiated food and autoclaved water. To overcome their resistance to CDI, all mice received an antibiotic pretreatment, as previously described^21^. Mice consumed an antibiotic mixture of kanamycin (0.4 mg/ml), gentamicin (0.035 mg/ml), colistin (850 U/ml), metronidazole (0.215 mg/ml) and vancomycin (0.045 mg/ml) in the drinking water for 3 days. After this treatment, mice were switched to regular autoclaved water. Two days later, all mice received a single dose of clindamycin (250 µg) by intraperitoneal injection (IP), 24 h prior to challenge. Mice were then infected by oral gavage with 10^5^ spores of *C. difficile* strains. Mice from the control group were pretreated with antibiotic but were orally gavaged with water. Animals were observed daily for signs of disease (diarrhea, hunched posture and ruffled fur). Two days after challenge, mice were euthanized and caecal tissues were collected, washed with PBS and cut in 3 pieces for further analyses (gene expression, histopathology and immunoblotting).

### Animal statements

For the mouse studies, animal care and animal experiments were carried out in strict accordance with the Committee for Research and ethical Issues of the International Association for the study of pain (IASP). The animal experimentation protocol was approved by the Animal Welfare Committee of the Paris-Sud University and animal experiments were performed according to the University Paris-Sud guidelines for the husbandry of laboratory animals.

### Fecal shedding of *C. difficile* during infection of mice

Unit forming colonies (UFC)/g feces of vegetative cells were obtained after serial dilution of feces in PBS and plating in BHI medium supplemented with 3% horse blood and Oxoid *C. difficile* supplement (250 mg/L D-cycloserine, 8 mg/L Cefoxitin). Plates were incubated in an anaerobic chamber (atmosphere of 90% N_2_, 5% CO_2_ and 5% H_2_) at 37 °C. For spore count, an alcoholic shock was performed and then samples were plated in the same medium supplemented with taurocholate 0.1%.

### Quantitative histologic assessment of inflammation in the caecum

The caecal segments assigned for histological studies were fixed in 10% neutral formalin for 18 h, transferred into 70% ethanol and paraffin embedded. 3 µm sections were stained with hematoxylin and eosin and analyzed in a blinded manner using a histopathological scoring scheme. Briefly, caecal inflammation was evaluated with the following criteria measured on 10 different microscopic fields: submucosal edema, degree of lamina propria mononuclear cell infiltration, epithelial damage as previously described^35^. We added the criteria of loss of goblet cells which was graded as follows: 0: >28; 1: 11–28; 2: 1–10; 3: <1 ^36^ (Supplementary Table S1).

### SDS-PAGE and Western blot analysis

Caecal tissue was disrupted and homogenized in a buffer containing 186 mM β-mercaptoethanol, 1% bromophenol blue, 10 mM NaF, 25 mM NaPPi, 1 mM Na3VO_4_, using a microtube adapted pellet mixer. After lysis, samples were incubated at 100 °C for 10 min and centrifuged to remove insoluble materials. Proteins were resolved by SDS–PAGE, and gels were transferred to polyvinylidene difluoride (PVDF) membrane (GE Healthcare). For immunoblotting, membranes were washed with TBS 0.1% Tween 20, blocked in TBS (0.1% Tween 20, 5% milk) and probed overnight with the following specific antibodies: anti-ERK1/2, anti-JNK, anti-IκBα, anti-phosphorylated (anti-p)ERK1/2 and anti-pJNK (Cell Signaling Technology), or anti-actin (Millipore). Blots were then incubated with HRP-linked secondary antibodies (Cell Signaling Technology), followed by chemiluminescence detection with the ECL Plus kit (Millipore) according to the manufacturer’s instructions. Chemiluminescence signals were detected with a Fusion FX (Vilber Lourmat) and analyzed densitometrically with Fusion-CAPT software (Vilber Lourmat).

### Quantitative real-time reverse transcription PCR (qRT-PCR)

Tissue from the caecum was submerged in RNA*later* RNA Stabilization Reagent (Qiagen) to protect the RNA. After stabilization for 15 min at room temperature, tissue was lysed using Lysing Matrix D and a FastPrep apparatus (MP Biomedicals). Total RNA was then isolated using the RNeasy Mini Kit (Qiagen). RNA quantification and quality were assessed by a 2100 Bioanalyzer Agilent. RNA was reverse-transcribed to first strand cDNA using the RT² First Strand Kit (Qiagen). cDNA was prepared from 1 µg RNA using SuperScript™ III Reverse Transcriptase (Invitrogen) with random primers as described by the manufacturer. qPCR was performed in a 10 µl reaction volume containing 4 ng of cDNA, 5 µl of SSo Advanced™ SYBR Green Supermix (Bio-Rad) and 500 nM gene-specific primers. The primers designed with Primer3 software, not to amplify genomic DNA, are listed in Supplementary Table S2. Reactions were run on a CFX96 Real-time system (Bio-Rad) with the following cycling conditions: 30 s polymerase activation at 95 °C and 40 cycles at 95 °C for 5 s and 60 °C for 10 s. An additional step from a start at 65 °C to 95 °C (0.5 °C/0.5 s) was performed to establish a melting curve in order to verify the specificity of the real-time PCR reaction for each primer pair. The results were normalized using the geometric averaging of the GAPDH as reference gene. Normalized relative quantities were calculated using the ΔΔCT method^37, 38^.

Data were collected from three independent experiments and expressed as down- or up-regulation fold. The statistical differences were determined by Student’s t-test. P values < 0.05 were considered statistically significant.

### Toxicity assay on Vero cell line

Vero kidney epithelial cell line (kindly donated by I. Beau, INSERM UMR-S 984, France) were grown in DMEM with L-glutamine (Invitrogen) supplemented with 10% heat-inactivated fetal calf serum at 37 °C in an atmosphere containing 5% CO_2_. Cell lines were harvested from the flask with trypsin (0.5 mg/ml)/EDTA (0.2 mg/ml), washed once with medium and seeded into culture plates (TPP, ATGC Biotechnologies) at the desired cell densities and incubated at 37 °C in an atmosphere containing 5% CO_2_ before experiments. Quantitative detection of toxin in fecal samples was performed by cytotoxicity assay. Briefly, feces from each mouse were collected and diluted in PBS at 1 mg/ml. After thorough mixing, the samples were centrifuged at 12000 g for 5 min, and the supernatants were collected and then sterilized through a 0.22 µm filter. Confluent Vero cells in 96-well plates were exposed to 1:2 to 1:262144 (vol/vol) dilutions of the supernatants in Dulbecco’s Modified Eagle Medium (DMEM) (Gibco) containing 10% heat-inactivated Fetal Bovine Serum (FBS) (Gibco). The plates were incubated at 37 °C in 5% CO_2_ and morphological changes were observed by microscopy after 24 h. The endpoint titers were expressed as the reciprocal of the highest dilution giving a 50% cytopathic effect (CPE). The assays were performed in triplicate on independent culture supernatants.

### Statistical analysis

Results from histologic assessment, fecal shedding, Western blot analysis, and toxicity assay are expressed as means ± sem. Different groups were compared by using Mann and Whitney test performed using StatEL software. P values < 0.05 were considered statistically significant.

## Electronic supplementary material

Supplementary information
