# Supplementary material for: Clostridium difficile flagella induce a pro-inflammatory response in intestinal epithelium of mice in cooperation with toxins
Source: Sci Rep. 2017 Jun 12;7:3256. doi: 10.1038/s41598-017-03621-z (PMC5468286; doi:10.1038/s41598-017-03621-z)
Supplement: Supplementary file 1 — Supplementary information [file 41598_2017_3621_MOESM1_ESM.pdf]

Supplementary data to:

Clostridium difficile flagella induce a pro-inflammatory response in intestinal epithelium of mice in cooperation with toxins

Authors

Jameel Batah[1], Hussein Kobeissy[1], Phuong Trang Bui Pham[1], Cécile Denève-Larrazet[1], Sarah Kuehne[2], Anne Collignon[1], Claire Janoir[1], Jean-Christophe Marvaud[1], Imad Kansau[1*]

[1]Faculté de Pharmacie, "Unité Bactéries Pathogènes et Santé" (UBaPS), Université Paris-Sud, Université Paris-Saclay, 92296 Châtenay-Malabry Cedex, France; [2]School of Dentistry. College of Medical and Dental Sciences. The University of Birmingham, Birmingham B5 7EG, UK.

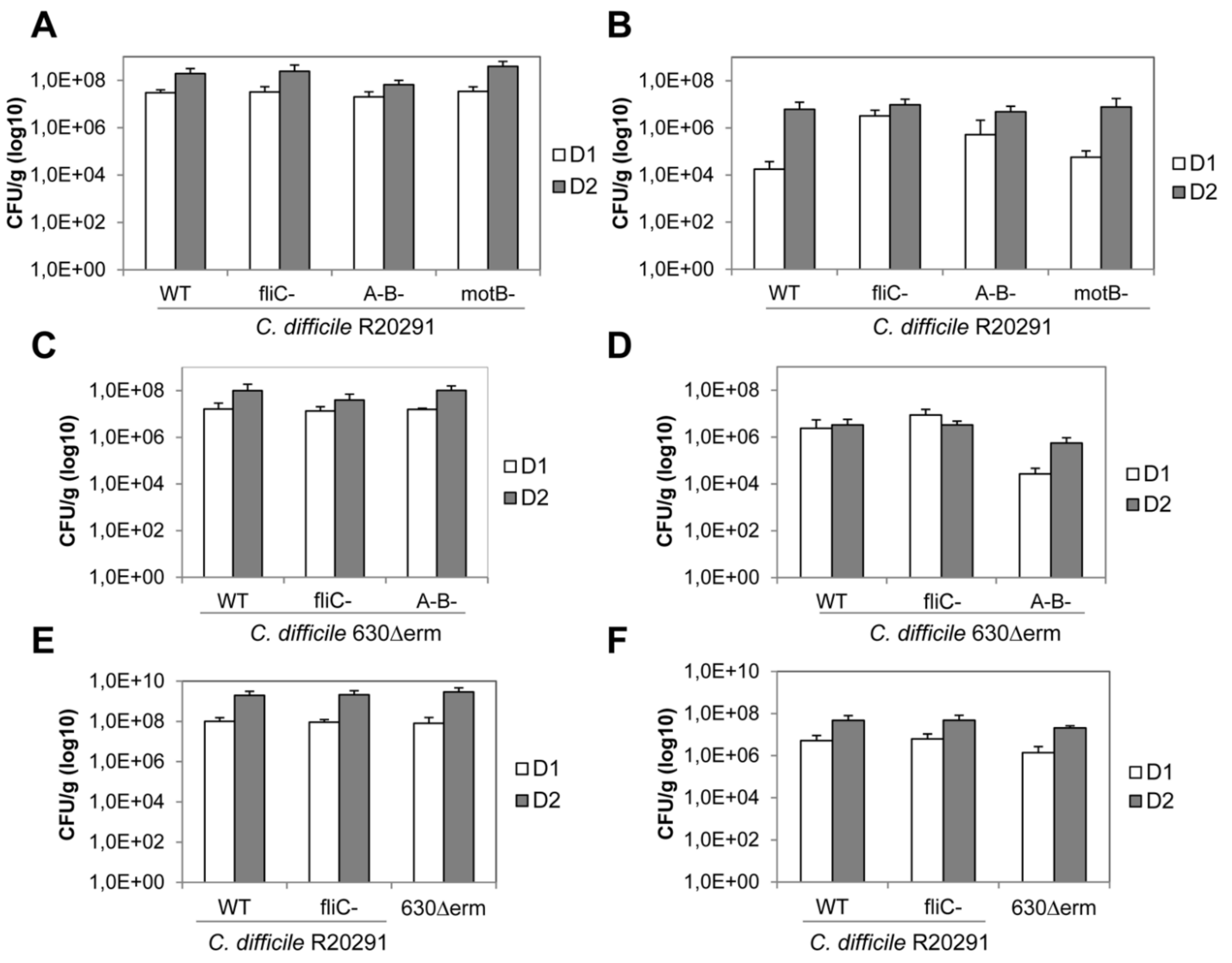

Figure S1. Fecal shedding of *C. difficile* strains during two days post-infection of mice. (A, C, E) vegetative cell count, (B, D, F) spore count. (A, B) R20291 WT strain and its respective *fliC* (ΔFliC), A$^-$B$^-$, and *motB* (MotB) mutants in conventional mice. (C, D) 630Δerm WT strain and its respective *fliC* (ΔFliC) and A$^-$B$^-$ mutants in conventional mice. (E, F) R20291 WT strain and its respective *fliC* (ΔFliC) mutant, and 630Δ*erm* WT in *tlr5*$^{-/-}$ KO mice. (A, C, E) UFC/g feces of vegetative cells were obtained after serial dilution of feces in PBS and plating in BHI medium supplemented with 3% horse blood and antibiotics. For spore count (B, D, F), an alcoholic shock was performed and then samples were plated in the same medium supplemented with taurocholate 0.1%. The results represent the average CFU/g of 10 mice for each group of animals infected with each strain at days 1 (D1) and 2 (D2) post-infection.

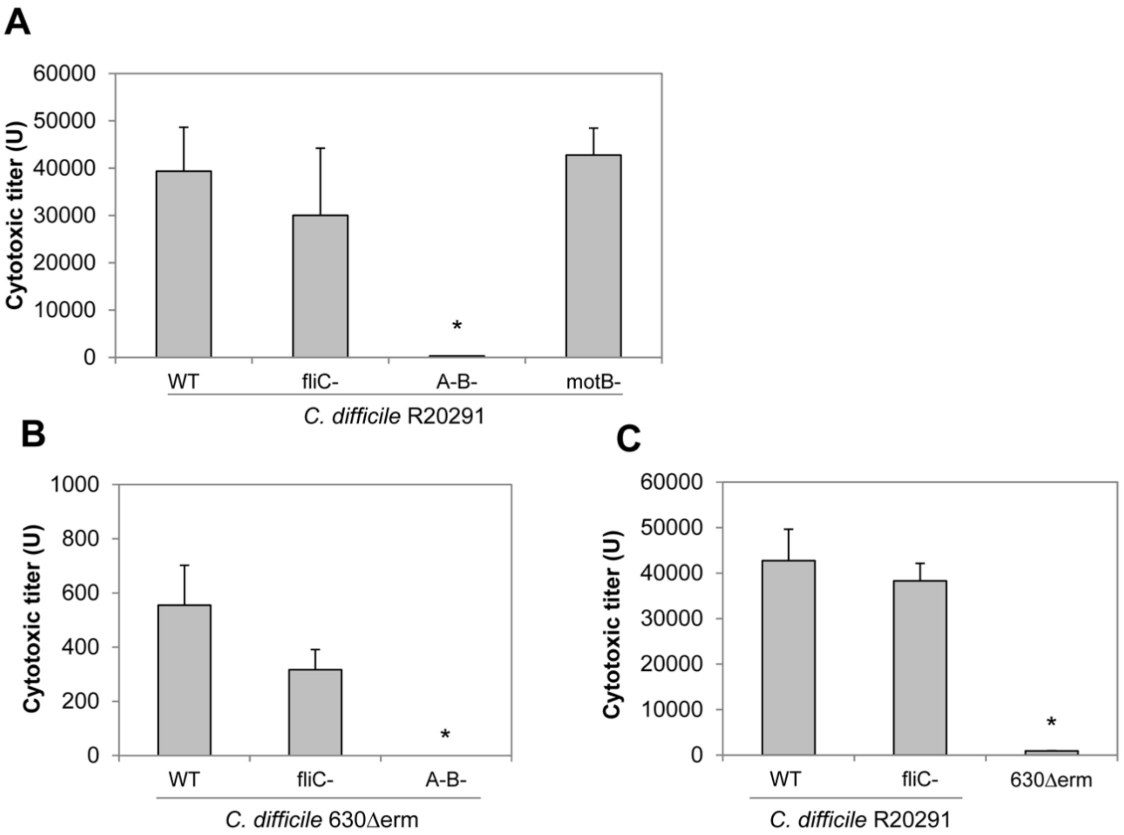

Figure S2. Cytotoxity activity in feces of *C. difficile* infected mice. (A) R20291 WT strain and its respective *fliC* (ΔFliC), A^-B^-, and *motB* (MotB) mutants in conventional mice. (B) 630Δ*erm* WT strain and its respective *fliC* (ΔFliC), and A^-B^- mutants in conventional mice. (C) R20291 WT strain and its respective *fliC* (ΔFliC) mutant, and 630Δ*erm* WT in *tlr5*$^{-/-}$ KO mice. Feces from mice were diluted in PBS and centrifuged. Supernatants were filtered (0.22 µm) and added from 1:2 to 1:262144 (vol/vol) dilutions to confluent Vero cells. Morphological changes were observed by microscopy after 24 h of incubation at 37°C in 5% CO_2. The endpoint titers were expressed as the reciprocal of the highest dilution giving a 50% cytopathic effect. The assays were performed in triplicate on independent culture supernatants. The bars represent the mean scores for each group of animals (n = 10) and standard deviations. * P <0.01 compared to WT-infected mice.

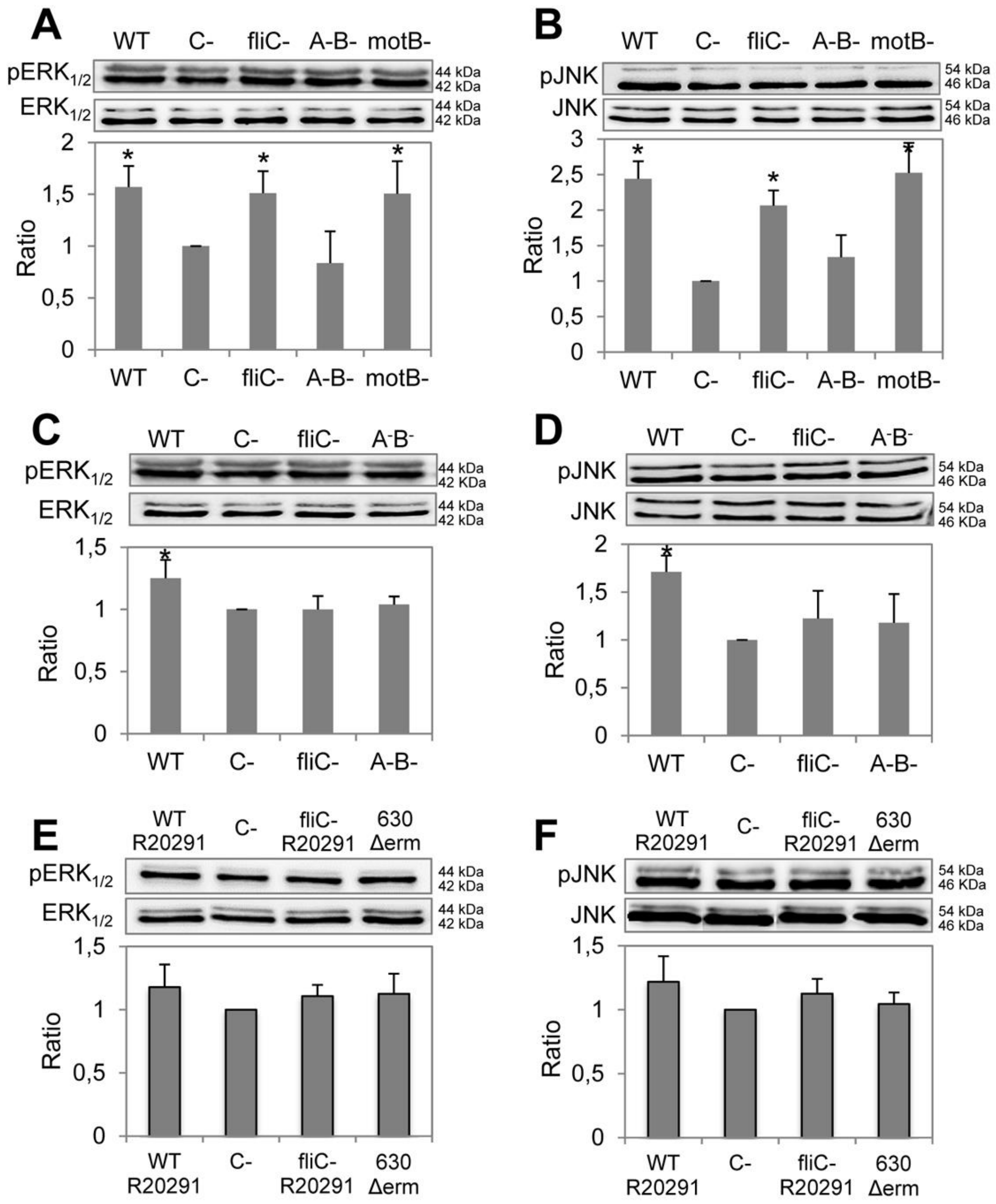

Figure S3. *C. difficile* flagella-induced phosphorylation of ERK and JNK. Caecal lysates were prepared as indicated in Material and Methods section and proteins were resolved by SDS-PAGE. (A, B) R20291 derivative-infected C57BL/6 mice; (C, D) 630Δ*erm* derivative-infected C57BL/6 mice, and (E, F) R20291 derivative- or 630Δ*erm* WT-infected C57BL/6 $tlr5^{-/-}$ KO mice. Western blots were then performed using (A, C, E) anti-phophoERK1/2, ERK1/2, or (B, D, F) anti-phophoJNK, JNK antibodies. Western blot cropped pictures (full-length blots are in the Supplementary Information file) show the results of a representative experiment. The density of the bands was measured using Fusion software. Ratios pERK/ERK (A C, E), and pJNK/JNK (B, D, F) were calculated and for the negative control (C-, uninfected mice), this ratio was normalized to 1. The ratio of the other samples was reported to the negative control. Results represent the mean (n = 10) ± standard deviations for each group of animals. * = Statistically significant differences (P <0.05) compared to the negative control.

Table S1. Inflammation score developed for the *C. difficile* infection mouse model.

**1. Submucosal edema**	
0	no edema
1	mild edema with minimal (< 2X) multifocal submucosal expansion
2	moderate edema with moderate (2–3X) multifocal submucosal expansion
3	severe edema with severe (> 3X) multifocal submucosal expansion
4	same as score 3 with diffuse submucosal expansion.
**2. Cellular infiltration (per 400x high power field)**	
0	no inflammation (0-5 cells)
1	minimal multifocal neutrophilic infiltration (6-20 cells)
2	moderate multifocal neutrophilic infiltration (greater submucosal involvement) (21-60 cells)
3	severe multifocal to coalescing neutrophilic infiltration (greater submucosal ± mural involvment (61-100 cells)
4	same as score 3 with abscesses or extensive mural involvement (>100 cells)
**3. Epithelial damage**	
0	no epithelial changes
1	minimal multifocal superficial epithelial damage. Desquamation (notable shedding of epithelial cells into the lumen)
2	moderate multifocal superficial epithelial damage. Mucosal erosion (loss of epithelium with retention of architecture or gaps of 1-10 cells)
3	severe multifocal epithelial damage (same as above) +/− pseudomembrane (intraluminal neutrophils, sloughed epithelium in a fibrinous matrix). Mucosal ulceration (destruction of lamina propria or gaps of >10 cells)
4	same as score 3 with significant pseudomembrane or epithelial ulceration (focal complete loss of epithelium)
**4. Loss of goblet cell (per 400X high power field)**	
0	>28
1	11-28
2	1-10
3	<1

Table S2. Primers used for individual qRT-PCR analysis.

Gene	Primers
mKC (IL-8)	F'-GCTGGGATTCACCTCAAGAA / R'-AGGTGCCATCAGAGCAGTCT
mIL-6	F'-CACAAAGCCAGAGTCCTTCAGAGA / R'-CTAGGTTTGCCGAGTAGATCT
mIL-1β	F'-ATGGCAACTGTTCCTGAACTCAACT / R'-CAGGACAGGTATAGATTCTTTCCTTT
m-IL22	F'- GCTCAGCTCCTGTCACATCA/ R'-GTTGAGCACCTGCTTCATCA
mCXCL10	F'-CCCACGTGTTGAGATCATTG / R'-GAGGCTCTCTGCTGTCCATC
mTNF-α	F'-AACTTTGGCATTGTGGAAGG / R'-ACACATTGGGGGTAGGAACA
mGAPDH	F'- GAACTGGCAGAAGAGGCACT / R'-AGGGTCTGGGCCATAGAACT

Anti-IκB-α Ab

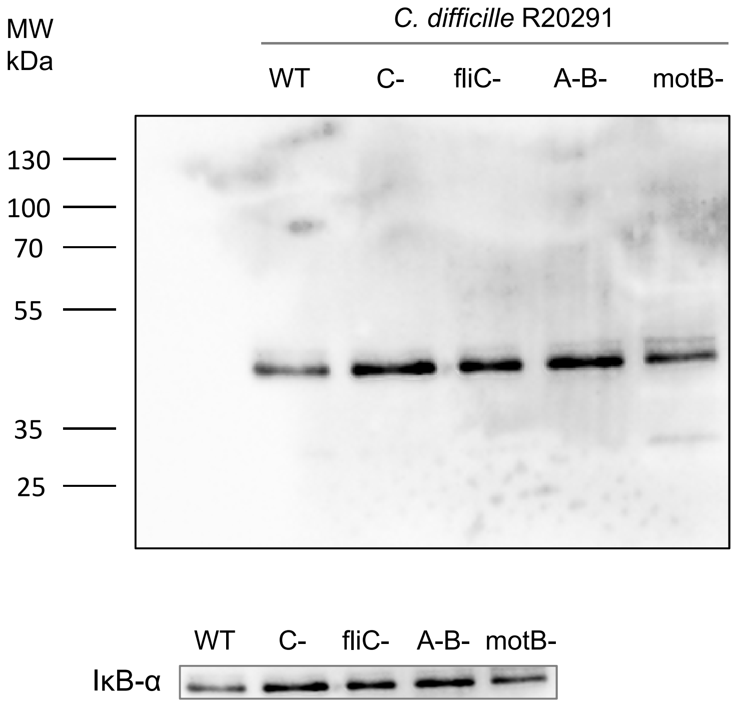

Cropped blot in
Figure 4A

Anti-actin Ab

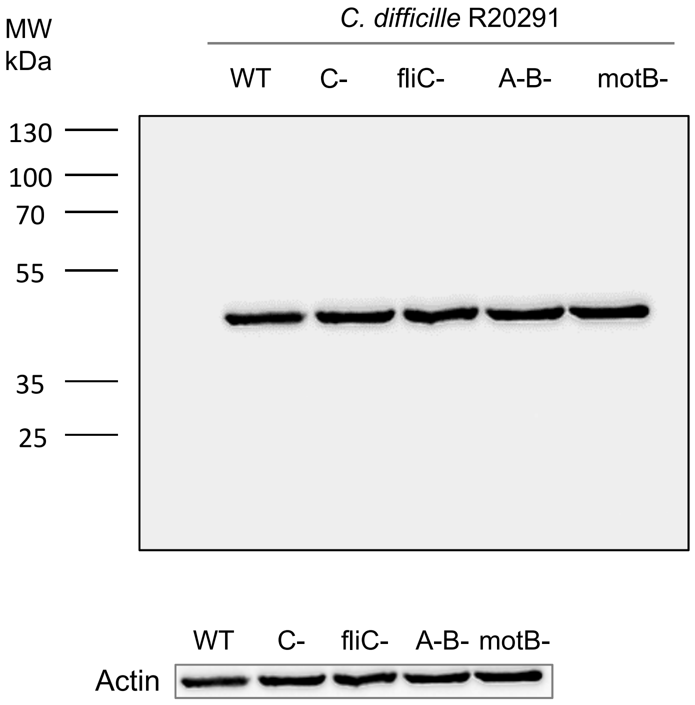

Cropped blot in
Figure 4A

Anti-IκB-α Ab

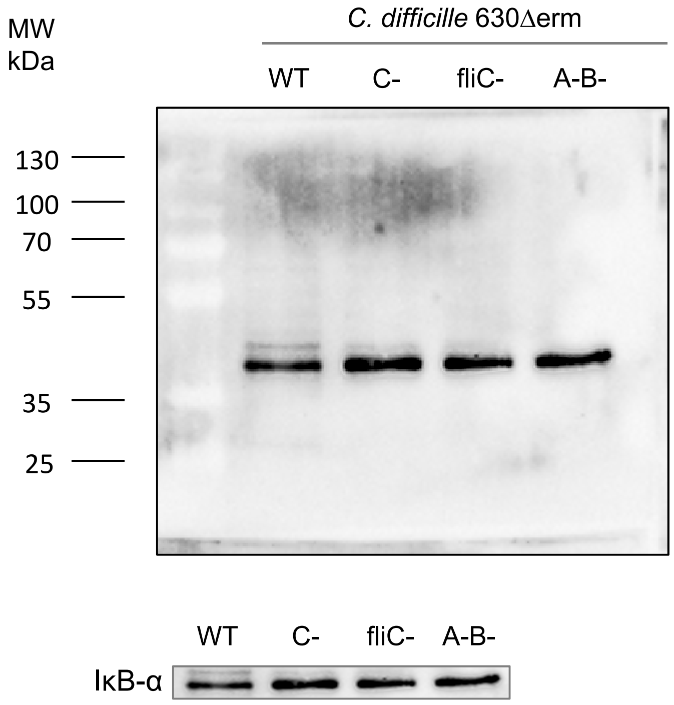

Cropped blot in Figure 4B

Anti-actin Ab

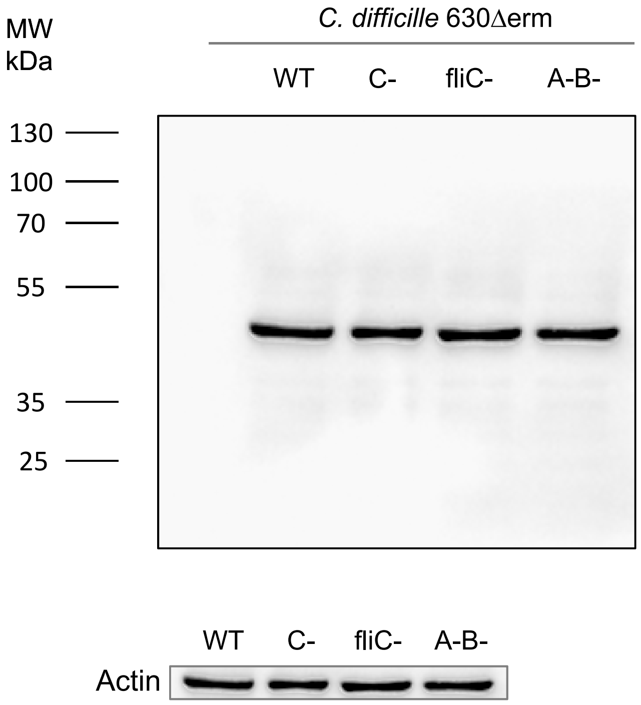

Cropped blot in Figure 4B

Anti-IκB-α Ab

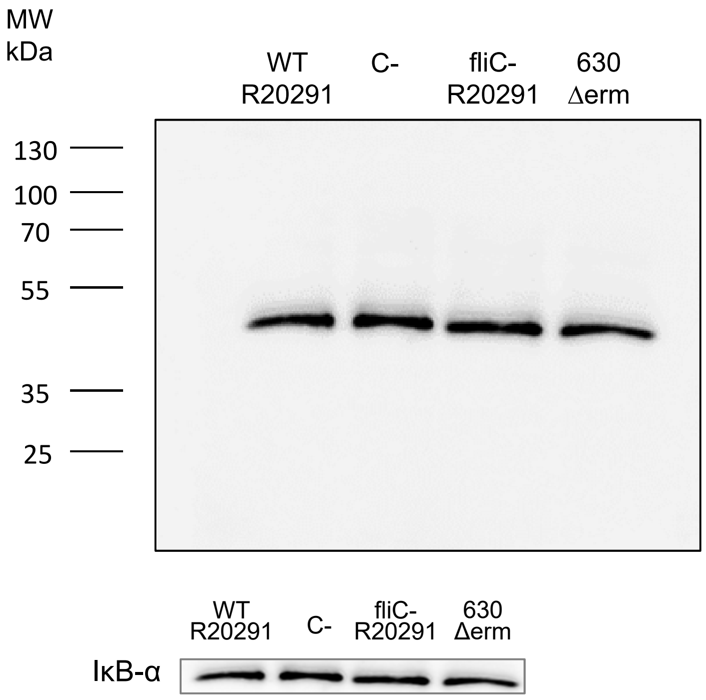

Cropped blot in Figure 4C

Anti-actin Ab

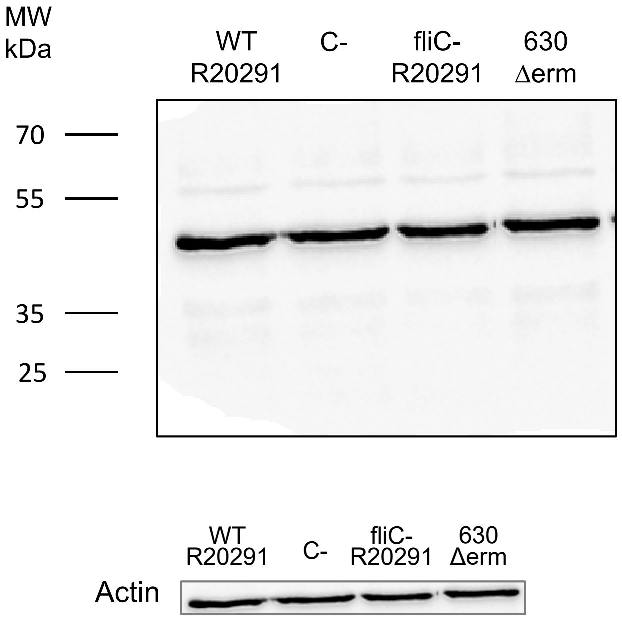

Cropped blot in Figure 4C

Anti-pERK Ab

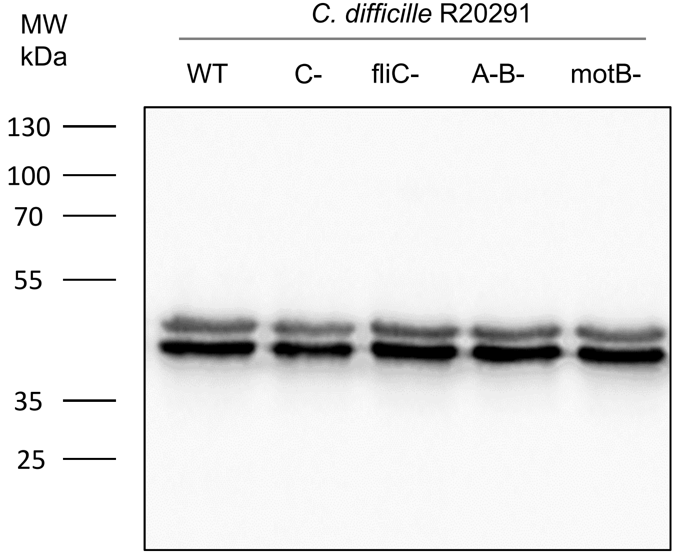

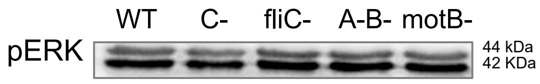

Cropped blot in
Suplementary Figure S3 A

Anti-ERK Ab

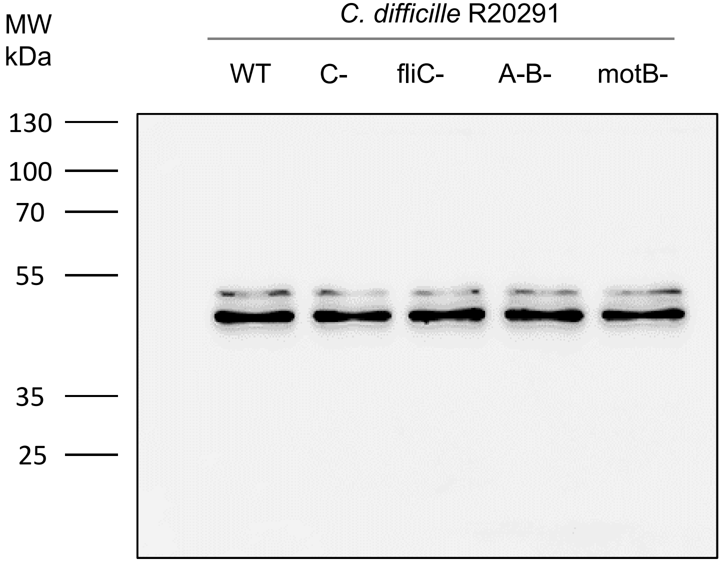

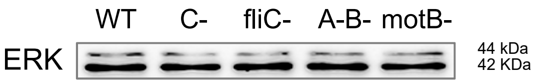

Cropped blot in
Suplementary Figure S3 A

Anti-pJNK Ab

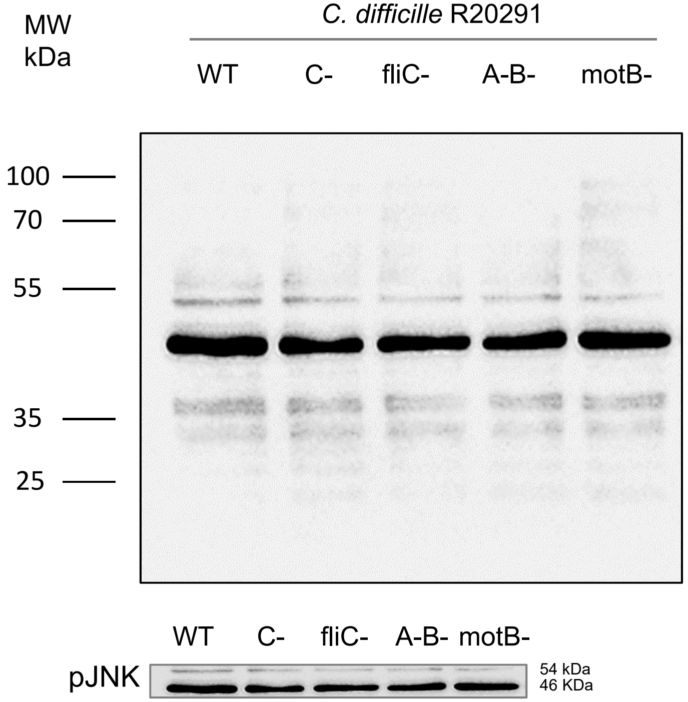

Cropped blot in
Suplementary Figure S3 B

Anti-JNK Ab

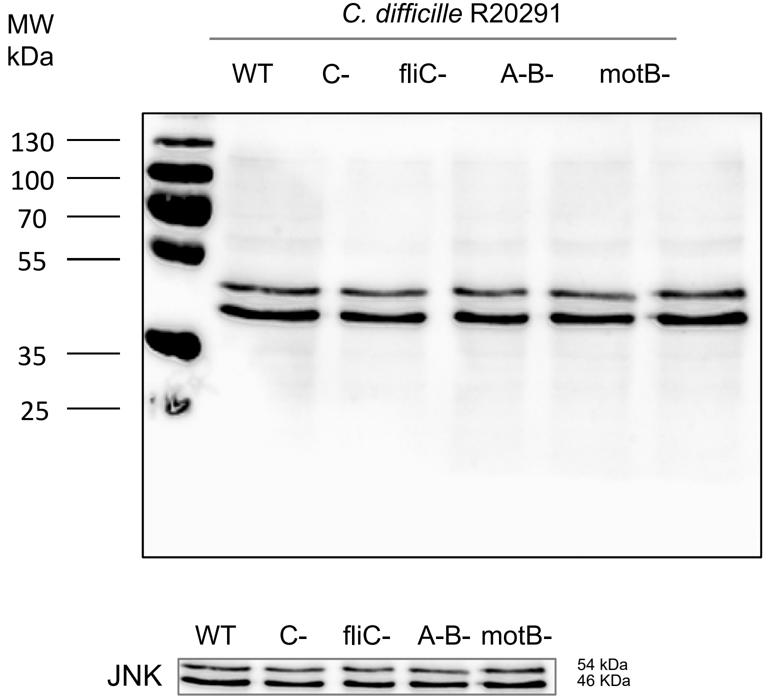

Cropped blot in
Suplementary Figure S3 B

Anti-pERK Ab

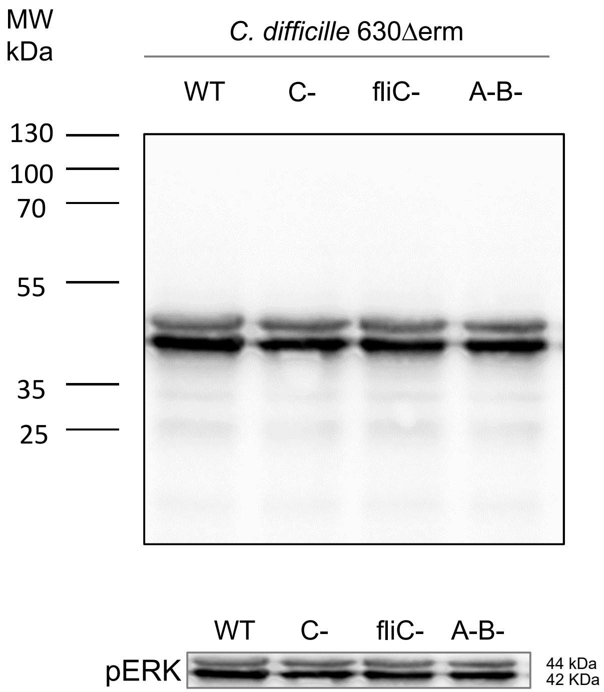

Cropped blot in
Suplementary Figure S3 C

Anti-ERK Ab

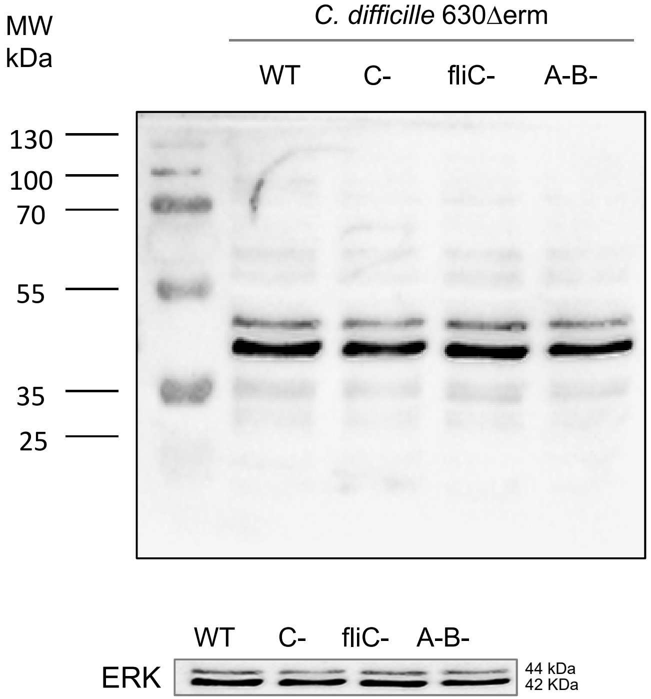

Cropped blot in
Suplementary Figure S3 C

Anti-pJNK Ab

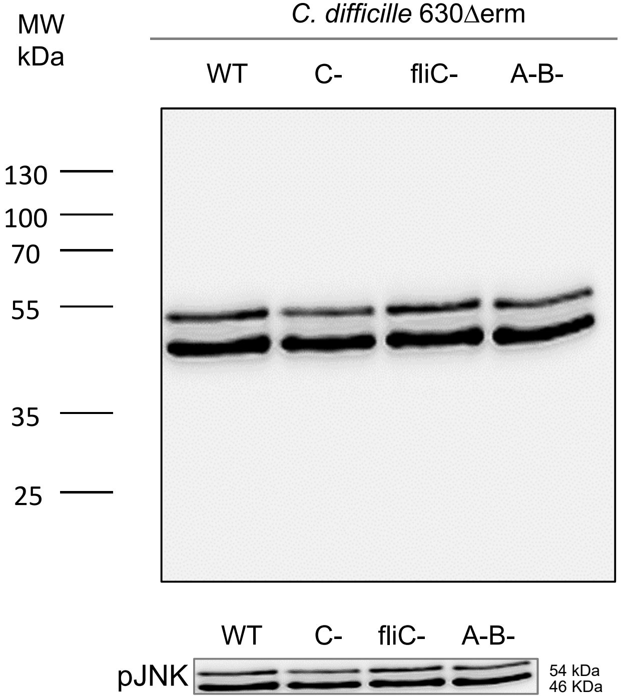

Cropped blot in
Suplementary Figure S3 D

Anti-JNK Ab

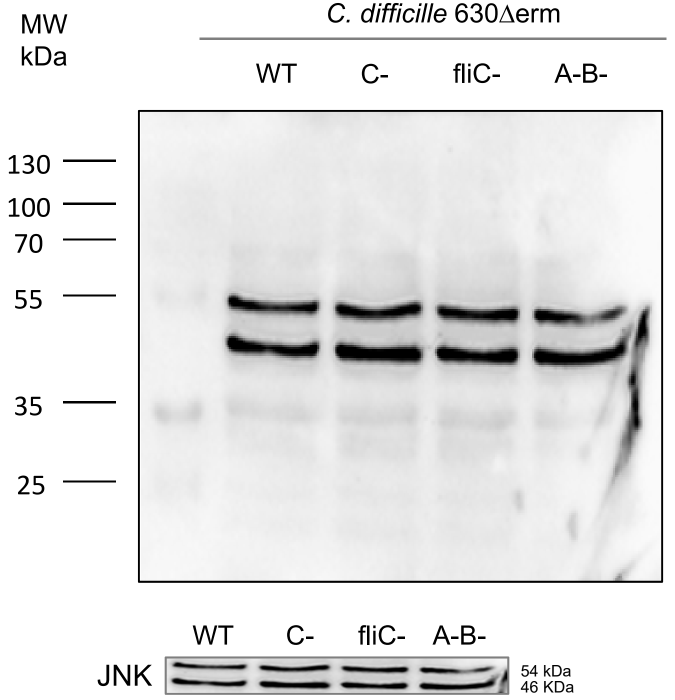

Cropped blot in
Suplementary Figure S3 D

Anti-pERK Ab

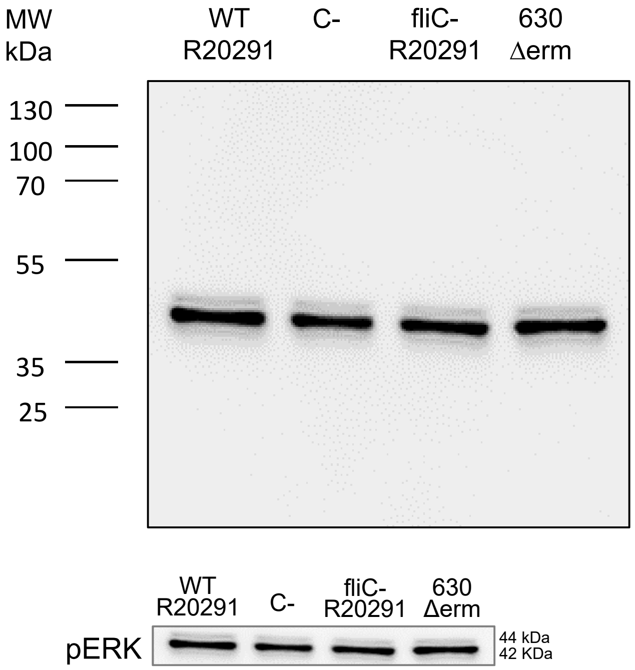

Cropped blot in
Suplementary Figure S3 E

Anti-ERK Ab

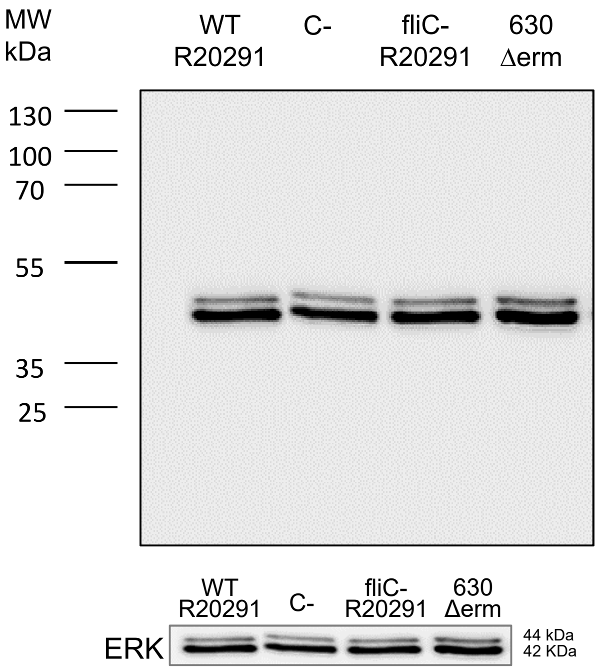

Cropped blot in
Suplementary Figure S3 E

Anti-pJNK Ab

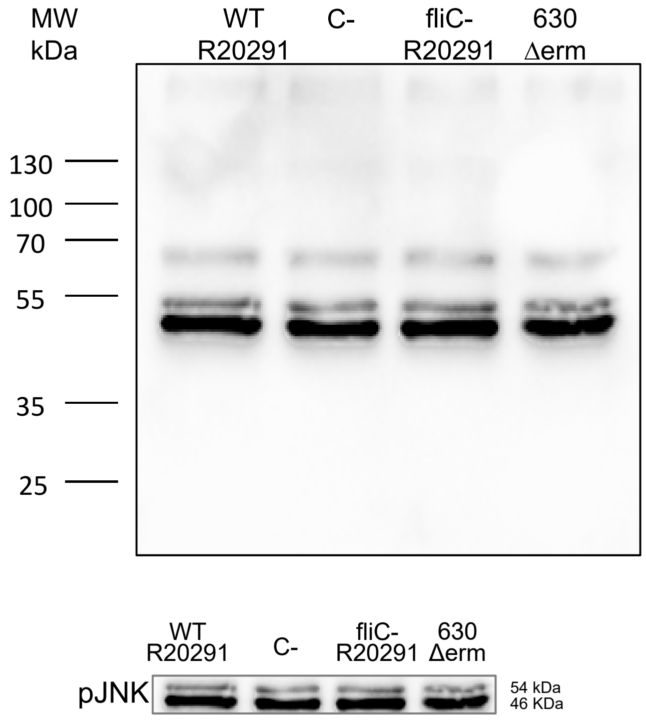

Cropped blot in
Suplementary Figure S3 D

Anti-JNK Ab

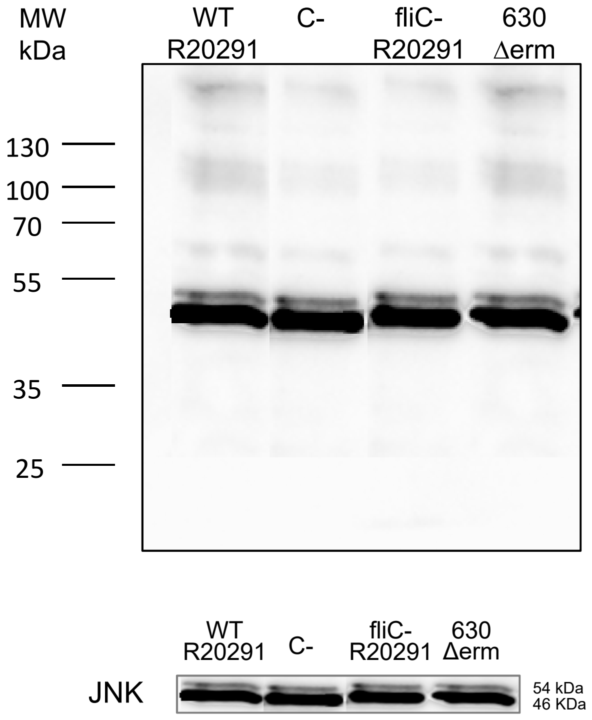

Cropped blot in
Suplementary Figure S3 D
